# Mitomycin-Induced Thrombotic Thrombocytopenic Purpura Treated Successfully With Plasmapheresis and Steroid: A Case Report

**DOI:** 10.7759/cureus.23525

**Published:** 2022-03-26

**Authors:** Misbahuddin Khaja, Zaheer A Qureshi, Sameer Kandhi, Faryal Altaf, Laura Yapor

**Affiliations:** 1 Internal Medicine/Pulmonary Critical Care, Icahn School of Medicine at Mount Sinai/BronxCare Health System, New York City, USA; 2 Internal Medicine, Icahn School of Medicine at Mount Sinai/BronxCare Health System, New York City, USA; 3 Internal Medicine, BronxCare Health System, New York City, USA; 4 Pulmonary and Critical Care, Icahn School of Medicine at Mount Sinai/BronxCare Health System, New York City, USA

**Keywords:** plasmapheresis, hematology, mitomycin, antitumor, thrombotic microangiopathies, chemotherapeutic, hus, hemolytic uremic syndrome, ttp, thrombotic thrombocytopenic purpura

## Abstract

Thrombotic thrombocytopenic purpura (TTP) is a thrombotic microangiopathy (TMA) caused by severely reduced ADAMTS13 or the von Willebrand factor-cleaving protease (VWFCP) enzyme resulting in low platelet and red blood cell counts along with severe renal, cardiac, and neurological dysfunction. Plasmapheresis is the treatment of choice. Mitomycin, a widely used chemotherapeutic agent for gastrointestinal (GI) cancers anal and breast cancers, has been reported to occasionally cause severe TTP and hemolytic uremic syndrome (HUS) cases. Here, we present a case of a 57-year-old African American transgender patient who presented with worsening kidney function, thrombocytopenia, and anemia following mitomycin therapy for her anal squamous cell carcinoma. Peripheral smear showed numerous schistocytes, and the patient was diagnosed with TTP because of low ADAMTS13 levels. The patient was started on plasmapheresis and steroid with ultimate improvement in condition. TTP is a rare condition that can be idiopathic or acquired. Further research is required to assess the complexity of the underlying mechanism. Early diagnosis and aggressive management often lead to a favorable outcome.

## Introduction

Thrombotic thrombocytopenic purpura (TTP) is a thrombotic microangiopathy (TMA) caused by severely reduced activity of ADAMTS13 or the von Willebrand factor-cleaving protease (VWFCP) enzyme resulting in low platelet count, low red blood cells, and often kidney heart, and brain dysfunction [1,2]. About one per 100,000 people are affected [2].

TTP is a medical emergency and form of microangiopathy in which ADAMTS13 is reduced and can be hereditary or acquired [3]. Its diagnosis involves the pentad of fever, microangiopathic hemolytic anemia (MAHA), thrombocytopenia, renal involvement, and neurologic involvement, but only 5% of cases involve the full pentad. Acquired or secondary TTP can be caused by cancer, infection, stem cell transplantation, organ transplantation, and medication such as chemotherapy.

Plasmapheresis is the usual treatment for TTP and often yields good results. Mitomycin is used as a chemotherapeutic agent because of its antitumor activity. It is widely used in gastrointestinal (GI), anal, and breast cancers [4]. Its side effects include bone marrow suppression, lung fibrosis, and renal damage [5].

## Case presentation

We present a case of a 57-year-old African American transgender patient who presented to the emergency department (ED) from her oncologist for worsening kidney function. Her medical history includes Human Immunodeficiency Virus (HIV) with a CD4 count of 470 cells/mm^3^ and undetectable viral load currently on antiretroviral treatment, hypertension, and anal cell carcinoma managed with chemotherapy (mitomycin), and daily radiation therapy. She has also been concomitantly taking hormonal therapy for her transition, including estrogen and spironolactone.

The patient was diagnosed with invasive squamous cell carcinoma, well to moderately differentiated with superficial ulceration and tumor focally extending to lateral margins of the ulcer. A follow-up fluorodeoxyglucose (FDG)-positron emission tomography (PET) scan of the whole body to evaluate local, regional disease/metastatic disease confirmed hypermetabolic activity confined to anus with extension to the intergluteal cleft; however, no nodal or metastatic disease activity was identified in the rest of the body, and the tumor was staged as IIB with a TNM Staging: T3N0M0. The patient was initiated on 5-fluorouracil 1000 mg/m² and mitomycin 10 mg/m². She tolerated her initial cycle of chemotherapy well. She continued to receive daily radiation cycles.

On presentation to the ED, the patient complained of worsening fatigue and weakness over the last six days, associated with decreased oral intake, secondary pain, difficulty swallowing, dysuria, lower abdominal pain, constipation, and difficulty in micturition. She was hemodynamically stable with a pulse rate of 87 beats per minute, blood pressure 135/94mmHg, temperature 98.2 F, oxygen saturation of 97% on room air. The complete physical exam was unremarkable. However, her labs showed severe neutropenia with an absolute neutrophil count of 100 cells/mm^3^, severe anemia, severe thrombocytopenia, elevated blood urea nitrogen, creatinine 5.5 mg/dl (baseline 0.9 mg/dl), and severely decreased serum bicarbonate with serum pH 7.04. Initial laboratory values are shown in Table 1.

**Table 1:** Initial laboratory values HGB: hemoglobin; INR: international normalized ratio; HCV: hepatitis C virus; RPR: rapid plasma reagin *QuantiFERON TB Gold, QIAGEN N.V., Venlo, Netherlands

Complete Blood Count	Results
WBC count	0.2 ( 4.8-10.8 k/uL)
Absolute Neutrophil Count	0.1 (1.5-8.0 k/ul)
RBC Count	2.74 ( 4.50-5.90 MIL/uL)
HGB	9.7 (12.0-16.0 g/dL)
Hematocrit	28.4 (42-51 %)
Platelet	63(150-400 k/uL)
Peripheral smear	Few schistocytes, few burr cells, markedly decreased platelet cells
General Coagulation	
Prothrombin Time	13.7 (9.9-13.3 seconds)
Partial Thromboplastin Time	27.8 (27.2-39.6 seconds)
INR	1.19 (0.85-1.14)
D-dimer Assay, Plasma	403 (0-230 ng/mL)
General Chemistry	
pH	7.044 (7.35-7.45)
Sodium, Serum	135 (135-145 mEq/L)
Potassium, Serum	4.6(3.5-5.0 mEq/L)
Bicarbonate , Serum	7 (24-30 mEq/L)
Blood Urea Nitrogen, Serum	64(8-26 mg/dL)
Creatinine, Serum	5.5( 0.5-1.5 mg/dL)
Hepatic Function Panel	
Bilirubin, Serum total	0.8 ( 0.2-1.1 mg/dL)
Bilirubin, Serum Direct-Conjugated	0.3(0.0-0.3 mg/dL)
Alkaline Phosphatase, Serum	84 (56-155 unit/L)
Aspartate Transaminase, Serum	89 (9-48 unit/L)
Alanine Aminotransferase, Serum	55 (5-40 unit/L)
Lactic acid Level	1.7 ( 0.5-1.6 mmoles/L)
Lactate Dehydrogenase, Serum	217 ( 110-210 unit/L)
Haptoglobin, Serum	225 ( 30-200 mg/dL)
Creatinine Kinase	1908 (20-200 units/L)
Lipase , serum	25 (<61 U/L)
Uric Acid , serum	13.0 (2.5-8 mg/dl)
C- reactive protein	336 (<5 mg/L)
Thyroid Stimulating Hormone	0.65 (0.40-4.50mIU/L)
Absolute CD4 count	470 (490-1740 cells/uL)
HIV Viral Load	Undetectable
Hepatitis B Core Total Antibody	Reactive
Hepatitis B Core IgM Antibody	Non-Reactive
Hepatitis B Surface Antibody	Reactive
Hepatitis B Surface Antigen	Non-Reactive
HCV Viral Load	Undetectable
RPR Screen	Non-Reactive
QuantiFERON TB Gold*	Negative
ADAMTS13 activity	0.55 (0.68-1.63 IU/mL)

**Figure 1:**
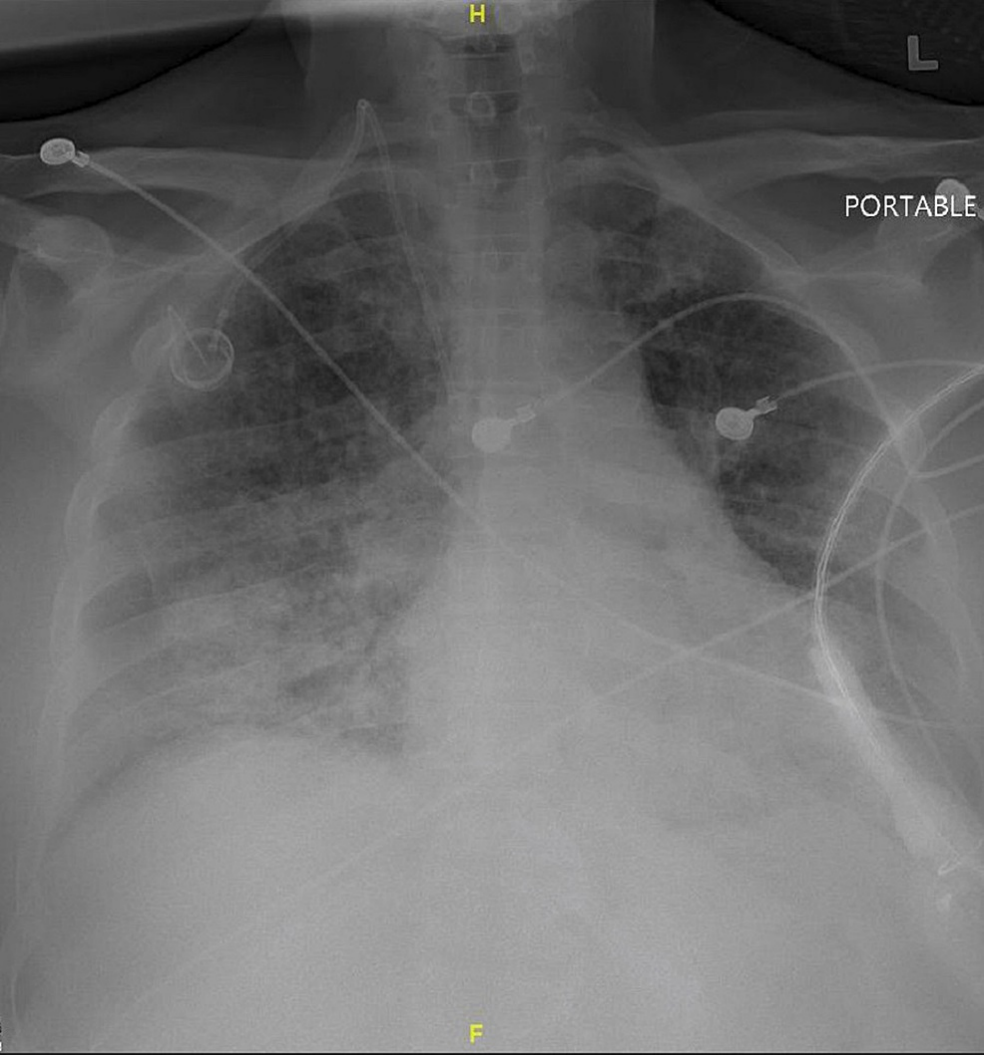
Chest x-ray showing bilateral infiltrates

**Figure 2:**
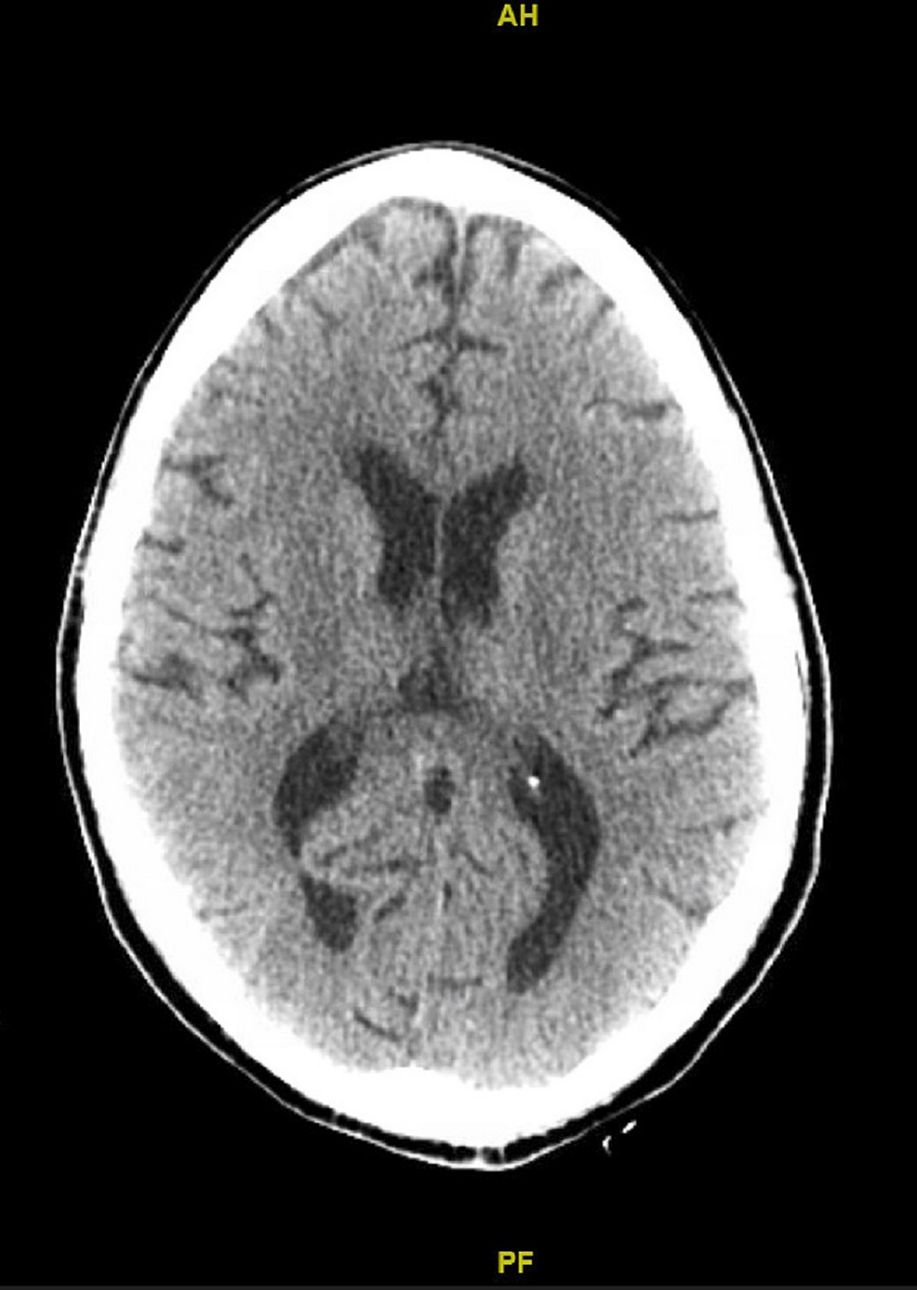
CT Head showing normal architecture

**Figure 3:**
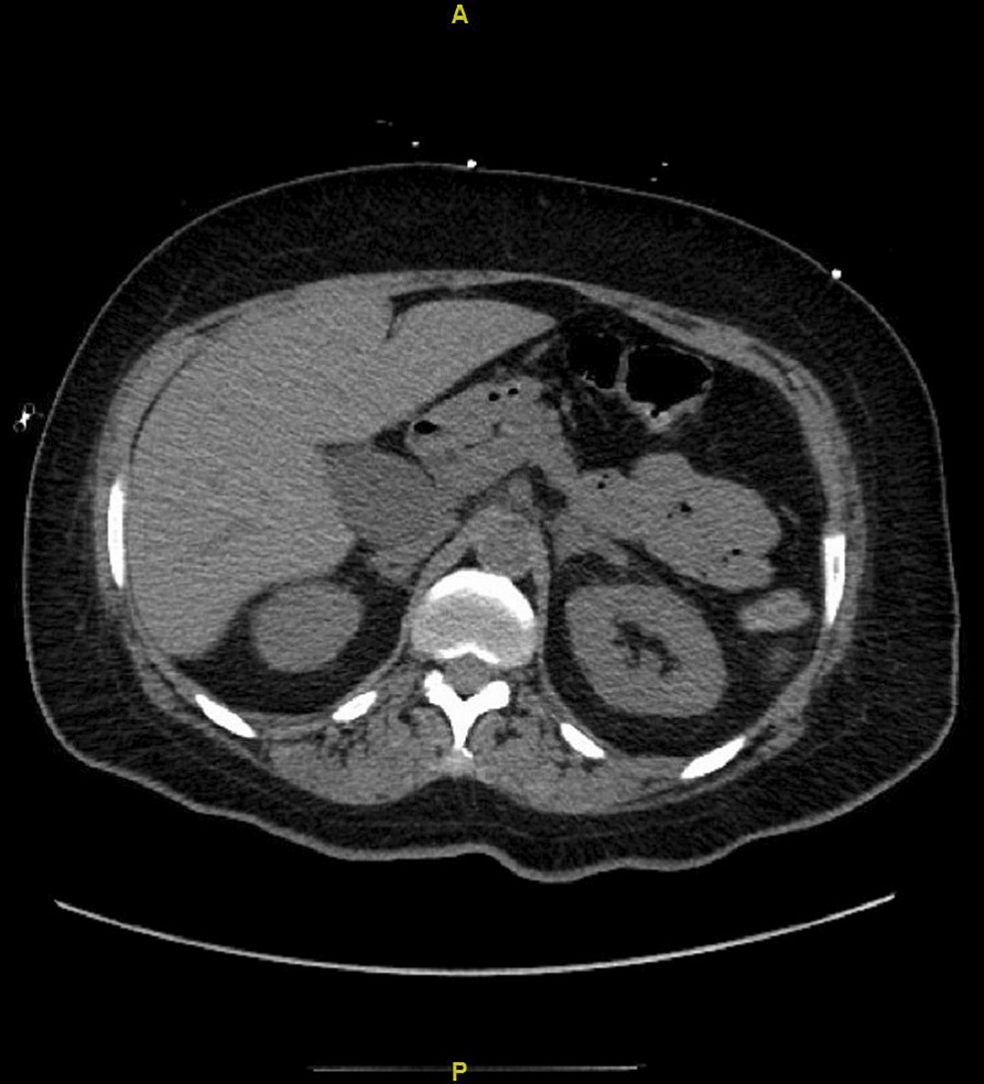
CT Abdomen and Pelvis showing normal architecture

The patient was admitted to the Intensive care unit (ICU) for acute kidney injury, complicating metabolic acidosis and electrolyte abnormalities into severe anion gap. Chest x-ray showed patchy consolidation on bilateral lung bases consistent with pneumonia, as shown in Figure 1. Computerized Tomography (CT) of the head and abdomen/pelvis were unremarkable, as shown in Figure 2 and Figure 3. Due to metabolic acidosis, the patient was aggressively resuscitated and started on a sodium bicarbonate drip. The patient was placed on reverse isolation given profound neutropenia and was empirically started on broad-spectrum antibiotics with pseudomonal coverage and daily subcutaneous filgrastim injections 480mcg/daily until absolute neutrophil count improves to >1000 cells/mm3. However, the patient eventually developed an acute drop in her hemoglobin to 5.4 grams/dl without any signs of overt bleeding, requiring packed RBC transfusions and severe sepsis with septic shock requiring vasopressor support.

Septic workups consisting of blood, urine, respiratory cultures were unrevealing. The patient eventually developed acute hypoxic respiratory failure secondary to volume overload from worsening renal failure, necessitating the need for noninvasive positive-pressure ventilation (NIPPV) and acute hemodialysis. In addition, hematology was consulted for an opinion on suspected TTP/hemolytic uremic syndrome (HUS) secondary to severe thrombocytopenia and renal failure and needed plasmapheresis.

**Figure 4:**
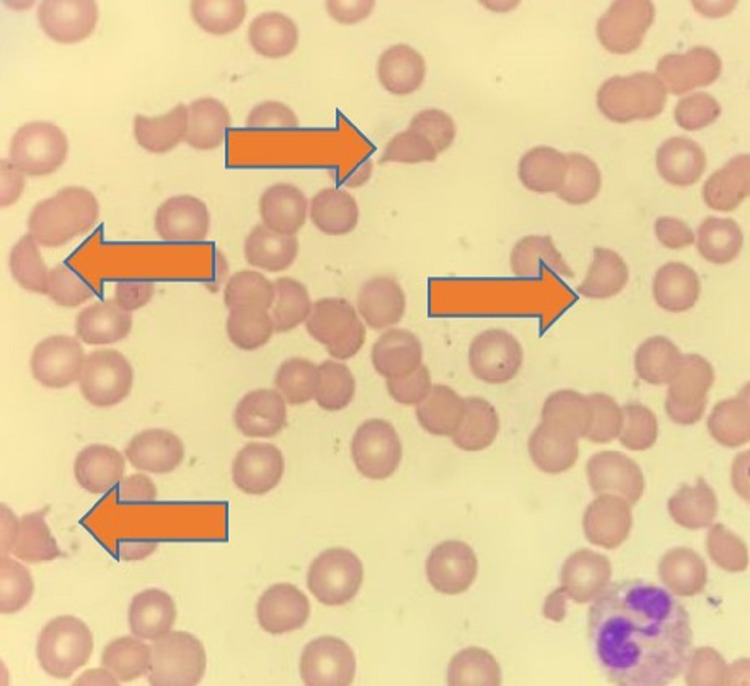
Peripheral smear showing schistocytes (orange arrows)

**Figure 5:**
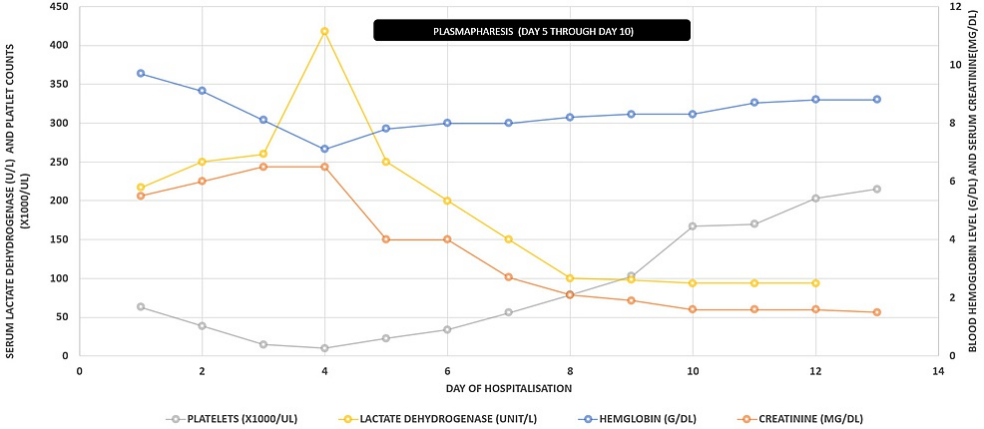
The trend of platelets, LDH, hemoglobin, and creatinine over the course of treatment with plasmapheresis LDH: lactate dehydrogenase

Differentials included bone marrow suppression causing thrombocytopenia, but mitomycin-related TTP could not be completely ruled out. Follow-up labs showed peripheral smear with few schistocytes, as shown in Figure 4. ADAMTS13 activity from admission was 0.55IU/ml (normal 0.68-1.63 IU/ml). The patient was started on plasmapheresis and dexamethasone 8mg twice daily and held off from radiation therapy for cancer till the acute issues were resolved. She was then empirically treated using fresh frozen plasma and underwent a plasmapheresis session. The patient received four sessions of plasmapheresis, following which her platelets, LDH, and haptoglobin gradually improved. Plasmapheresis was stopped following the improvement of platelet count to 100,000/mm^3^ but suggested to continue with steroid therapy and gradually taper. The patient was taken off hemodialysis following improvement in serum blood urea nitrogen to 15 mg/dl and serum creatinine to 1.4mg/dl. The patient has eventually titrated off vasopressors and bi-level positive pressure ventilation and transferred out of the ICU to the regular medicine floor. The trend of platelets, lactate dehydrogenase (LDH), hemoglobin, and creatinine throughout treatment with plasmapheresis is presented in Figure 5.

## Discussion

TTP was first described by Eli Moschcowitz in 1924 [6]. It is a form of thrombotic microangiopathy characterized by systemic microvascular platelet aggregation and RBC destruction. About one per 100,000 people are affected. Onset is usually in adulthood, and women are more often affected [2]. It is characterized by small vessel platelet-rich thrombi that cause thrombocytopenia, microangiopathic hemolytic anemia, and organ damage. It is a medical emergency and fatal if not treated promptly. However, with appropriate treatment, survival rates are 90%. In children, the possibility of hereditary TTP must be considered. In adults, immune TTP is more common. The classic presentation of TTP includes pentad of clinical signs and symptoms, including thrombocytopenia, microangiopathic hemolytic anemia, neurological abnormalities, renal failure, and fever [6].

TTP has diverse etiologies, including cancer, infection, stem cell transplantation, organ transplantation, and medication such as chemotherapy. Drug-related TTP is common and represents 12% of cases [7]. TTP can be divided into hereditary and acquired. It can result from ADAMTS13 deficiency. In the acquired form, many things can trigger the formation of antibodies against ADAMTS13. Drugs that can cause TTP include antibiotics, oral contraceptive pills (OCPS), opioids, and chemotherapeutic agents such as mitomycin and immunomodulators [8].

Plasma exchange or plasmapheresis is the treatment of choice. Before the plasma therapy, most of the patients with TTP did not survive [9,10]. Immunosuppressants such as steroids and rituximab can be used while platelet transfusion has no role in treatment [11,12]. Test for deficiency of ADAMTS13 is 100% sensitive and specific for the diagnosis of TTP. The presence of ADAMTS13 inhibitor suggests the acquired forms of TTP [13]. Current guidelines from the Association for the Advancement of Blood & Biotherapies and the American Society for Apheresis recommend daily plasma exchange with replacement of 1-1.5 times the predicted plasma volume of the patient [14,15].

The mechanism involving autoantibody-mediated inhibition of ADAMTS13 is responsible for cleaving multimers of von Willebrand factor (vWF) into smaller units. These multimers of vWF increase platelet adhesions of endothelial injury areas, resulting in small platelet clots called thrombi. This leads to a decrease in circulating platelets, which cause life-threatening bleeding. Erythrocytes passing these clots are subject to stress resulting in damage to their membranes and rupture, microangiopathic hemolytic anemia, and schistocyte formation. In addition, the presence of these blood flow to organs is reduced due to these clots leading to cellular and end-organ damage [2].

The platelet count; combined hemoLysis variable; absence of active cancer; absence of stem-cell or solid-organ transplant; mean corpuscular volume (MCV); international normalized ratio (INR); creatinine (PLASMIC) score can be easily calculated using the findings available on presentation and can predict the levels of ADAMTS13 activity [16]. It is composed of the following criteria: a platelet count < 30,000/μL, evidence of hemolysis (reticulocyte count > 2.5%, elevated indirect bilirubin > 2 mg/dL, undetectable haptoglobin levels), MCV < 90 fl, INR < 1.5, and creatinine < 2 mg/dl with no active cancer, solid organ or stem cell transplant [17]. This scoring system gives one point for each of the above criteria. A score of 0-4 suggests a low probability of TTP, a score of 5 suggests an intermediate probability of TTP, and a score of 6-7 suggests a high probability of TTP. A 2020 systematic review and meta-analysis confirmed the diagnostic accuracy of the PLASMIC score in individuals with suspected TTP [18]. This score cannot substitute for clinical judgment and should not be used to confirm or exclude TTP diagnosis. It can help guide the decision to start therapy while waiting for the results of ADAMTS13 activity.

Mitomycin C is used as a chemotherapeutic agent because of its antitumor activity. It is widely used in GI, anal, and breast cancers [4]. However, its side effects include bone marrow suppression, lung fibrosis, and renal damage [5]. Mitomycin induces TTP by endothelial injury, which is dose-dependent [19]. Usually, the onset of TTP results after four to nine weeks of completion of chemotherapy with mitomycin, but symptoms may occur up to 15 months later [20]. One possible mechanism of mitomycin-induced TTP is believed to be suppression of the generation of ADAMTS13 inhibitor. Mitomycin rarely causes TTP or HUS; however, it is important to remain vigilant because cytopenias can easily be misunderstood to myelosuppression.

Further research is needed to understand how mitomycin or other anticancer drugs causes TTP or HUS. Internists and intensivists should be aware of this rare complication and periodically screen the patients undergoing treatment. To the best of our knowledge, there are very few reported cases of mitomycin-induced TTP. It is essential to remember this association, as cancer patients are widely exposed to mitomycin and other anticancer drugs.

## Conclusions

Mitomycin remains an integral part of treating GI, especially squamous cell anal cancer. It rarely causes TTP or HUS; however, the physician should remain vigilant of these side effects. Patients should be made aware of the potential medication side effects before the onset of the therapy. Early diagnosis and prompt treatment of mitomycin-induced TTP can prevent devastating consequences. Plasmapheresis and steroids remain the first-line option, but the use of rituximab can be considered in cases that are refractory to the initial therapy. Though there have been only a very few reported cases of mitomycin-induced TTP in literature, it is imperative to remember this association, as cancer patients are widely exposed to mitomycin and other anticancer drugs
